# Supplementary material for: Resistance to ceftazidime–avibactam and other new β-lactams in Pseudomonas aeruginosa clinical isolates: a multi-center surveillance study
Source: Microbiol Spectr. 2024 Jun 27;12(8):e04266-23. doi: 10.1128/spectrum.04266-23 (PMC11302676; doi:10.1128/spectrum.04266-23)
Supplement: Supplemental material — Tables S1 to S3. [file spectrum.04266-23-s0001.pdf]

Table S1. Antibiotic susceptibility profiles of the 120 *P. aeruginosa* isolates included in the study.

Strain	yr of isolation	Centre code	Origin	MIC (mg/L) / Inhibition zone diameter (mm)																											
				AMK (mg/L)	AMK (S/I/R)	ATM (mg/L)	ATM (S/I/R)	FEP (mg/L)	FEP (S/I/R)	CAZ (mg/L)	CAZ (S/I/R)	CZA (mg/L)	CZA (S/I/R)	C/T (mg/L)	C/T (S/I/R)	CST (mg/L)	CST (S/I/R)	IMP (mg/L)	IMP (S/R/I)	I-R (mg/L)	I-R (S/I/R)	MEM (mg/L)	MEM (S/I/R)	MVB (mg/L)	MVB (S/I/R)	TZP (mg/L)	TZP (S/I/R)	TOB (mg/L)	TOB (S/I/R)	FDC (mm)	FDC (S/R)
PSA1	2022	C2	HAP/VAP	32	R	>32	R	>16	R	>32	R	>16	R	>8	R	2	S	>8	R	8	R	>16	R	>16	R	32	R	>4	R	18	R
PSA2	2022	C2	HAP/VAP	<=2	S	32	R	16	R	>32	R	4	S	2	S	1	S	>8	R	2	S	8	I	8	S	>32	R	<=0.5	S	18	R
PSA3	2022	C2	UTI	8	S	32	R	>16	R	>32	R	>16	R	>8	R	1	S	>8	R	>8	R	>16	R	>16	R	>32	R	>4	R	25	S
PSA4	2022	C2	UTI	32	R	8	I	16	R	32	R	>16	R	>8	R	2	S	>8	R	>8	R	>16	R	>16	R	32	R	>4	R	32	S
PSA5	2022	C2	HAP/VAP	8	S	32	R	16	R	8	I	8	S	4	S	2	S	>8	R	4	R	16	R	8	S	>32	R	<=0.5	S	25	S
PSA6	2022	C2	HAP/VAP	4	S	32	R	4	I	2	I	8	S	1	S	1	S	>8	R	2	S	4	I	4	S	16	I	<=0.5	S	23	S
PSA7	2022	C2	HAP/VAP	16	S	>32	R	>16	R	>32	R	>16	R	>8	R	1	S	>8	R	8	R	>16	R	>16	R	>32	R	>4	R	8	R
PSA8	2022	C2	HAP/VAP	>32	R	16	I	8	I	>32	R	>16	R	>8	R	1	S	>8	R	>8	R	>16	R	>16	R	>32	R	>4	R	27	S
PSA9	2023	C2	UTI	>32	R	32	R	>16	R	>32	R	16	R	>8	R	1	S	>8	R	8	R	16	R	16	R	>32	R	>4	R	27	S
PSA10	2023	C2	UTI	>32	R	>32	R	>16	R	32	R	>16	R	>8	R	1	S	4	I	2	S	16	R	16	R	>32	R	>4	R	21	R
PSA11	2023	C2	HAP/VAP	8	S	32	R	>16	R	32	R	8	S	4	S	1	S	8	R	0.5	S	8	I	8	S	>32	R	1	S	22	S
PSA12	2023	C2	HAP/VAP	<=2	S	8	I	2	I	2	I	2	S	2	S	1	S	<=1	I	0.5	S	0.5	S	0.5	S	8	I	<=0.5	S	24	S
PSA13	2023	C2	UTI	<=2	S	16	I	4	I	2	I	4	S	0.5	S	1	S	<=1	I	0.5	S	2	S	1	S	8	I	<=0.5	S	23	S
PSA14	2023	C2	BSI	>32	R	8	I	16	R	16	R	16	R	>8	R	2	S	>8	R	>8	R	>16	R	>16	R	32	R	>4	R	28	S
PSA15	2023	C2	BSI	<=2	S	>32	R	16	R	>32	R	8	S	2	S	1	S	<=1	I	0.5	S	0.5	S	0.12	S	>32	R	<=0.5	S	27	S
PSA16	2023	C2	HAP/VAP	>32	R	16	I	16	R	16	R	<=0.25	S	4	S	1	S	>8	R	>8	R	>16	R	>16	R	>32	R	>4	R	28	S
PSA17	2023	C2	HAP/VAP	<=2	S	32	R	8	I	2	I	8	S	1	S	1	S	<=1	I	0.5	S	2	S	2	S	16	I	<=0.5	S	25	S
PSA18	2023	C2	HAP/VAP	<=2	S	16	I	16	R	32	R	2	S	1	S	2	S	<=1	I	0.5	S	0.25	S	0.12	S	>32	R	<=0.5	S	23	S
PSA19	2023	C2	HAP/VAP	4	S	8	I	4	I	2	I	4	S	1	S	1	S	2	I	0.5	S	<=0.12	S	<=0.06	S	32	R	<=0.5	S	24	S
PSA20	2023	C2	UTI	<=2	S	4	I	<=1	I	2	I	1	S	0.5	S	1	S	<=1	I	0.25	S	<=0.12	S	<=0.06	S	8	I	<=0.5	S	26	S
PSA21	2023	C2	UTI	<=2	S	4	I	2	I	2	I	2	S	1	S	1	S	<=1	I	0.5	S	0.5	S	0.25	S	16	I	<=0.5	S	25	S
PSA22	2023	C2	BSI	4	S	8	I	2	I	2	I	2	S	0.5	S	2	S	<=1	I	0.5	S	0.25	S	0.5	S	4	I	<=0.5	S	28	S
PSA23	2023	C2	BSI	<=2	S	8	I	8	I	2	I	2	S	1	S	1	S	>8	R	2	S	4	I	4	S	8	I	<=0.5	S	30	S
PSA24	2023	C2	BSI	<=2	S	16	I	4	I	2	I	4	S	1	S	2	S	2	I	0.25	S	0.5	S	0.5	S	8	I	<=0.5	S	23	S
PSA25	2023	C2	UTI	<=2	S	-	-	16	R	8	I	8	S	4	S	-	-	1	I	-	-	0.25	S	-	-	>32	R	<=0.5	S	23	S
PSA26	2023	C2	UTI	4	S	8	I	2	I	2	I	4	S	1	S	1	S	<=1	I	0.5	S	0.25	S	0.25	S	16	I	<=0.5	S	29	S
PSA27	2023	C2	UTI	4	S	16	I	16	R	>32	R	4	S	2	S	1	S	<=1	I	0.5	S	<=0.12	S	<=0.06	S	>32	R	<=0.5	S	25	S
PSA28	2023	C2	BSI	>32	R	>32	R	16	R	2	I	4	S	>8	R	2	S	2	I	1	S	1	S	1	S	32	R	>4	R	25	S
PSA29	2023	C2	BSI	16	S	16	I	2	I	2	I	2	S	0.5	S	1	S	<=1	I	0.5	S	<=0.12	S	0.12	S	<=4	I	2	S	34	S
PSA30	2023	C2	UTI	4	S	4	I	2	I	2	I	2	S	0.5	S	1	S	<=1	I	0.5	S	0.25	S	0.12	S	<=4	I	<=0.5	S	27	S
PSA31	2023	C2	HAP/VAP	<=2	S	4	I	2	I	2	I	2	S	0.5	S	2	S	2	I	0.5	S	<=0.12	S	<=0.06	S	<=4	I	<=0.5	S	24	S
PSA32	2023	C2	UTI	8	S	32	R	16	R	8	I	4	S	2	S	1	S	2	I	0.5	S	0.5	S	0.25	S	>32	R	1	S	25	S
PSA33	2023	C2	UTI	4	S	8	I	2	I	2	I	2	S	0.5	S	2	S	<=1	I	0.5	S	0.25	S	0.12	S	8	I	<=0.5	S	25	S
PSA34	2023	C2	UTI	4	S	8	I	2	I	2	I	2	S	1	S	2	S	<=1	I	0.5	S	<=0.12	S	<=0.06	S	<=4	I	<=0.5	S	29	S

PSA35	2023	C2	BSI	4	S	4	I	2	I	2	I	2	S	0.5	S	1	S	4	I	0.5	S	0.5	S	0.25	S	8	I	<=0.5	S	28	S
PSA36	2023	C2	BSI	4	S	2	I	8	I	-	I	1	S	1	S	1	S	<=1	I	0.5	S	0.5	S	0.25	S	8	I	1	S	24	S
PSA37	2023	C2	HAP/VAP	<=2	S	32	R	4	I	2	I	4	S	1	S	2	S	>8	R	8	R	8	I	8	S	32	R	<=0.5	S	27	S
PSA38	2022	C5	HAP/VAP	32	R	>32	R	16	R	>32	R	16	R	>8	R	2	S	<=1	I	0.5	S	2	S	1	S	8	I	<=0.5	S	21	R
PSA39	2022	C5	HAP/VAP	16	S	>32	R	>16	R	>32	R	>16	R	>8	R	2	S	>8	R	>8	R	>16	R	>16	R	>32	R	>4	R	26	S
PSA40	2022	C5	HAP/VAP	32	R	-	-	16	R	>32	R	16	R	>16	R	2	S	>8	R	-	-	16	R	-	-	>32	R	1	S	29	S
PSA41	2022	C5	BSI	32	R	>32	R	>16	R	>32	R	>16	R	>8	R	1	S	>8	R	>8	R	>16	R	>16	R	>32	R	>4	R	26	S
PSA42	2023	C5	BSI	<=2	S	4	I	<=1	I	2	I	1	S	0.5	S	1	S	<=1	I	0.5	S	1	S	0.5	S	32	R	<=0.5	S	27	S
PSA43	2023	C5	BSI	4	S	8	I	2	I	2	I	2	S	0.5	S	1	S	2	I	0.5	S	0.5	S	0.5	S	<=4	I	<=0.5	S	29	S
PSA44	2023	C5	HAP/VAP	8	S	<=1	I	<=1	I	2	I	1	S	0.5	S	1	S	<=1	I	0.25	S	<=0.12	S	<=0.06	S	<=4	I	<=0.5	S	30	S
PSA45	2023	C5	HAP/VAP	4	S	16	I	4	I	2	I	4	S	0.5	S	1	S	<=1	I	0.5	S	1	S	1	S	8	I	<=0.5	S	29	S
PSA46	2023	C5	HAP/VAP	<=2	S	4	I	4	I	2	I	2	S	0.5	S	1	S	<=1	I	0.5	S	<=0.12	S	0.12	S	<=4	I	<=0.5	S	28	S
PSA47	2023	C5	HAP/VAP	4	S	32	R	16	R	2	I	4	S	2	S	1	S	<=1	I	0.5	S	1	S	1	S	>32	R	<=0.5	S	28	S
PSA48	2023	C5	HAP/VAP	8	S	8	I	4	I	2	I	2	S	1	S	1	S	<=1	I	0.5	S	0.25	S	0.25	S	<=4	R	<=0.5	S	28	S
PSA49	2022	C4	BSI	32	R	8	I	16	R	32	R	>16	R	>8	R	1	S	>8	R	>8	R	>16	R	>16	R	32	I	>4	R	30	S
PSA50	2022	C4	BSI	4	S	32	R	16	R	8	I	8	S	2	S	2	S	>8	R	2	S	16	R	16	R	>32	R	>4	R	27	S
PSA51	2022	C4	BSI	32	R	16	I	16	R	16	R	>16	R	>8	R	2	S	>8	R	>8	R	>16	R	>16	R	32	R	>4	R	28	S
PSA52	2022	C4	BSI	<=2	S	4	I	2	I	2	I	2	S	0.5	S	1	S	<=1	I	0.5	S	<=0.12	S	0.12	S	<=4	I	<=0.5	S	25	S
PSA53	2022	C4	BSI	4	S	8	I	2	I	2	I	2	S	0.5	S	1	S	<=1	I	0.5	S	<=0.12	S	<=0.06	S	<=4	I	<=0.5	S	28	S
PSA54	2022	C4	BSI	4	S	4	I	2	I	2	I	2	S	0.5	S	1	S	<=1	I	0.5	S	<=0.12	S	<=0.06	S	8	I	<=0.5	S	28	S
PSA55	2022	C4	BSI	4	S	8	I	2	I	2	I	2	S	0.5	S	2	S	2	I	0.25	S	<=0.12	S	<=0.06	S	<=4	I	<=0.5	S	30	S
PSA56	2023	C4	BSI	<=2	S	4	I	2	I	2	I	2	S	1	S	1	S	<=1	I	0.5	S	<=0.12	S	<=0.06	S	8	I	<=0.5	S	26	S
PSA57	2023	C4	BSI	8	S	2	I	2	I	2	I	1	S	0.5	S	1	S	2	I	0.5	S	<=0.12	S	<=0.06	S	<=4	I	<=0.5	S	25	S
PSA58	2023	C4	BSI	4	S	8	I	4	I	2	I	2	S	0.5	S	1	S	<=1	I	0.5	S	<=0.12	S	0.12	S	16	I	<=0.5	S	25	S
PSA59	2023	C4	BSI	4	S	4	I	2	I	2	I	4	S	0.5	S	1	S	<=1	I	0.5	S	0.25	S	0.25	S	<=4	I	<=0.5	S	27	S
PSA60	2023	C4	BSI	<=2	S	8	I	2	I	2	I	4	S	0.5	S	1	S	>8	R	2	S	4	I	2	S	<=4	I	<=0.5	S	33	S
PSA61	2023	C4	BSI	<=2	S	32	R	4	I	2	I	4	S	1	S	2	S	2	I	2	S	4	I	4	S	16	I	<=0.5	S	27	S
PSA62	2023	C4	BSI	32	R	16	I	16	R	16	R	16	R	>8	R	2	S	>8	R	>8	R	>16	R	>16	R	32	R	>4	R	27	S
PSA63	2022	C4	HAP/VAP	<=2	S	4	I	2	I	2	I	2	S	1	S	1	S	>8	R	2	S	4	I	4	S	<=4	I	<=0.5	S	33	S
PSA64	2022	C4	HAP/VAP	<=2	S	4	I	2	I	8	I	1	S	0.5	S	1	S	8	R	1	S	4	I	4	S	16	I	<=0.5	S	30	S
PSA65	2022	C4	HAP/VAP	4	S	4	I	4	I	2	I	2	S	0.5	S	1	S	2	I	0.5	S	<=0.12	S	<=0.06	S	<=4	I	<=0.5	S	29	S
PSA66	2022	C4	HAP/VAP	4	S	32	R	16	R	32	R	4	S	2	S	1	S	2	I	0.5	S	1	S	1	S	>32	R	<=0.5	S	28	S
PSA67	2022	C4	HAP/VAP	8	S	>32	R	16	R	>32	R	0.5	S	4	S	1	S	<=1	I	0.25	S	0.5	S	0.12	S	>32	R	<=0.5	S	28	S
PSA68	2022	C4	HAP/VAP	<=2	S	4	I	2	I	2	I	1	S	0.5	S	1	S	<=1	I	0.5	S	1	S	0.5	S	<=4	I	<=0.5	S	25	S
PSA69	2023	C4	HAP/VAP	4	S	8	I	4	I	2	I	2	S	0.5	S	1	S	<=1	I	0.5	S	0.25	S	0.5	S	<=4	I	<=0.5	S	24	S
PSA70	2023	C4	HAP/VAP	>32	R	8	I	>16	R	>32	R	>16	R	>8	R	2	S	>8	R	>8	R	>16	R	>16	R	>32	R	>4	R	28	S
PSA71	2023	C4	HAP/VAP	16	S	8	I	16	R	16	R	16	R	>8	R	1	S	>8	R	>8	R	>16	R	>16	R	16	I	>4	R	24	S
PSA72	2023	C4	HAP/VAP	8	S	8	I	8	I	16	R	16	R	>8	R	1	S	>8	R	>8	R	>16	R	>16	R	32	R	>4	R	24	S

PSA73	2022	C4	UTI	32	R	16	I	16	R	16	R	>16	R	>8	R	2	S	>8	R	>8	R	>16	R	>16	R	32	R	>4	R	27	S
PSA74	2023	C4	UTI	<=2	S	4	I	2	I	2	I	2	S	0.5	S	1	S	8	R	1	S	2	S	2	S	<=4	I	<=0.5	S	30	S
PSA75	2023	C4	UTI	4	S	4	I	4	I	2	I	2	S	1	S	1	S	2	I	0.5	S	0.5	S	0.25	S	16	I	<=0.5	S	28	S
PSA76	2023	C4	UTI	8	S	4	I	4	I	2	I	2	S	1	S	2	S	4	I	1	S	2	S	2	S	8	I	>4	R	29	S
PSA77	2023	C4	UTI	<=2	S	4	I	2	I	2	I	1	S	0.5	S	1	S	<=1	I	0.5	S	0.5	S	0.25	S	8	I	<=0.5	S	25	S
PSA78	2023	C4	HAP/VAP	<=2	S	32	R	8	I	2	I	8	S	1	S	1	S	>8	R	4	R	16	R	16	R	16	I	<=0.5	S	25	S
PSA79	2022	C1	HAP/VAP	4	S	4	I	2	I	-	I	0.5	S	1	S	1	S	<=1	I	0.5	S	0.5	S	0.5	S	<=4	I	1	S	22	S
PSA80	2022	C1	BSI	<=2	S	32	R	8	I	16	R	8	S	1	S	1	S	2	I	0.5	S	0.25	S	0.25	S	32	R	<=0.5	S	25	S
PSA81	2022	C1	BSI	<=2	S	8	I	4	I	4	I	2	S	0.5	S	1	S	2	I	0.25	S	<=0.12	S	<=0.06	S	8	I	<=0.5	S	28	S
PSA82	2022	C1	BSI	<=2	S	16	I	4	I	-	I	4	S	2	S	1	S	<=1	I	0.122	S	0.25	S	0.25	S	16	I	<=0.5	S	27	S
PSA83	2022	C1	BSI	<=2	S	32	R	16	R	16	R	8	S	2	S	1	S	<=1	I	0.25	S	0.5	S	0.25	S	>32	R	<=0.5	S	25	S
PSA84	2023	C1	HAP/VAP	4	S	<=1	I	2	I	-	I	<=0.25	S	0.5	S	2	S	4	I	0.5	S	0.25	S	0.12	S	<=4	I	<=0.5	S	24	S
PSA85	2023	C1	BSI	8	S	-	-	8	I	16	R	4	S	-	S	1	S	-	-	-	-	>16	R	-	-	32	R	-	-	29	S
PSA86	2022	C1	HAP/VAP	<=2	S	-	-	4	I	2	I	1	S	0.5	S	<=0.5	S	-	-	-	-	<=0.12	S	-	-	<=4	I	-	-	33	S
PSA87	2023	C1	HAP/VAP	<=2	S	-	-	8	I	2	I	2	S	0.5	S	<=0.5	S	-	-	-	-	<=0.12	S	-	-	8	I	-	-	30	S
PSA88	2022	C1	BSI	<=2	S	32	R	16	R	>16	R	4	S	4	S	1	S	8	R	-	-	0.5	S	1	S	>32	R	1	S	28	S
PSA89	2022	C1	HAP/VAP	<=2	S	-	-	8	I	8	I	4	S	1	S	<=0.5	S	-	-	-	-	4	I	-	-	>32	R	-	-	29	S
PSA90	2023	C1	BSI	<=2	S	-	-	2	I	1	I	1	S	0.5	S	<=0.5	S	-	-	-	-	<=0.12	S	-	-	4	I	-	-	25	S
PSA91	2023	C1	BSI	<=2	S	-	-	2	I	4	I	4	S	1	S	1	S	-	-	-	-	0.25	S	-	-	4	I	-	-	28	S
PSA92	2022	C1	BSI	<=2	S	-	-	>16	R	>32	R	4	S	>8	R	-	-	-	-	-	-	>16	R	-	-	>32	R	-	-	27	S
PSA93	2022	C1	BSI	32	R	4	I	16	R	>16	R	<=0.25	S	>8	R	-	S	>8	R	-	-	2	S	1	S	<=4	I	-	-	27	S
PSA94	2022	C1	BSI	<=2	S	-	-	8	I	8	I	4	S	2	S	1	S	-	-	-	-	16	R	-	-	16	I	-	-	27	S
PSA95	2022	C1	BSI	<=2	S	4	I	2	I	8	I	1	S	0.5	S	1	S	2	I	-	-	1	S	1	S	<=4	I	-	-	25	S
PSA96	2022	C1	BSI	<=2	S	32	R	16	R	>16	R	16	R	>8	R	1	S	>8	R	-	-	16	R	16	R	16	I	-	-	29	S
PSA97	2022	C1	BSI	<=2	S	-	-	2	I	2	I	4	S	4	S	<=0.5	S	-	-	-	-	0.5	S	-	-	16	I	-	-	24	S
PSA98	2022	C1	BSI	<=2	S	8	I	4	I	8	I	2	S	0.5	S	1	S	2	I	-	-	<=0.25	S	1	S	8	I	-	-	25	S
PSA99	2022	C1	BSI	<=2	S	8	I	2	I	2	I	2	S	0.5	S	1	S	2	I	-	-	1	S	1	S	<=4	I	-	-	28	S
PSA100	2022	C1	BSI	<=2	S	8	I	2	I	4	I	2	S	1	S	1	S	2	I	-	-	0.25	S	1	S	<=4	I	-	-	28	S
PSA101	2022	C1	BSI	<=2	S	8	I	2	I	>16	R	2	S	1	S	1	S	2	I	-	-	1	S	1	S	<=4	I	-	-	29	S
PSA102	2023	C1	BSI	<=2	S	-	-	4	I	4	I	-	S	-	S	-	-	2	I	-	-	<=0.25	S	-	-	8	I	1	S	33	S
PSA103	2022	C1	BSI	<=2	S	-	-	4	I	2	I	-	S	-	S	<=0.5	S	1	I	-	-	0.25	S	-	-	<=4	I	-	-	27	S
PSA104	2022	C1	BSI	<=2	S	-	-	4	I	4	I	-	S	-	S	<=0.5	S	1	I	-	-	<=0.25	S	-	-	8	I	-	-	23	S
PSA105	2022	C1	BSI	<=2	S	-	-	4	I	4	I	-	S	-	S	<=0.5	S	>8	R	-	-	16	R	-	-	32	R	-	-	27	S
PSA106	2022	C1	BSI	<=2	S	-	-	4	I	4	I	-	S	-	S	<=0.5	S	2	I	-	-	0.5	S	-	-	8	I	-	-	28	S
PSA107	2022	C1	BSI	<=2	S	-	-	4	I	4	I	-	S	-	S	<=0.5	S	2	I	-	-	1	S	-	-	<=4	I	-	-	29	S
PSA108	2022	C1	BSI	<=2	S	-	-	2	I	4	I	-	S	-	S	<=0.5	S	2	I	-	-	<=0.25	S	-	-	8	I	-	-	25	S
PSA109	2022	C1	BSI	8	S	-	-	8	I	8	I	-	S	-	S	<=0.5	S	8	R	-	-	8	I	-	-	8	I	-	-	28	S
PSA110	2022	C1	BSI	16	S	-	-	16	R	>8	R	16	R	-	R	<=0.5	S	>8	R	-	-	16	R	-	-	32	R	-	-	28	S

PSA111	2022	C1	BSI	<=2	S	-	-	4	I	4	I	-	S	-	S	<=0.5	S	2	I	-	-	0.5	S	-	-	8	I	-	-	27	S
PSA112	2022	C1	BSI	<=2	S	-	-	4	I	2	I	-	S	-	S	<=0.5	S	8	R	-	-	2	S	-	-	8	I	-	-	29	S
PSA113	2022	C1	BSI	<=2	S	-	-	8	I	8	I	-	S	-	S	<=0.5	S	>8	R	-	-	16	R	-	-	16	I	-	-	24	S
PSA114	2022	C1	BSI	<=2	S	-	-	<=1	I	<=0.5	I	-	S	-	S	<=0.5	S	4	I	-	-	<=0.25	S	-	-	<=4	I	-	-	26	S
PSA115	2022	C1	BSI	<=2	S	-	-	4	I	4	I	-	S	-	S	<=0.5	S	2	I	-	-	0.5	S	-	-	8	I	-	-	28	S
PSA116	2022	C1	BSI	<=2	S	-	-	4	I	4	I	-	S	-	S	<=0.5	S	1	I	-	-	<=0.25	S	-	-	<=4	I	-	-	30	S
PSA117	2022	C1	BSI	<=2	S	-	-	2	I	2	I	-	S	-	S	<=0.5	S	2	I	-	-	1	S	-	-	<=4	I	-	-	29	S
PSA118	2022	C1	BSI	<=2	S	-	-	4	I	2	I	-	S	-	S	<=0.5	S	2	I	-	-	1	S	-	-	8	I	-	-	29	S
PSA119	2023	C3	BSI	4	S	2	I	2	I	1	I	1	S	0.5	S	1	S	<=1	I	1	S	1	S	0.5	S	<=4	I	<=0.5	S	25	S
PSA120	2023	C3	HAP/VAP	16	S	>32	R	>16	R	>32	R	16	R	>8	R	1	S	8	R	4	R	>16	R	16	R	>32	R	>4	R	22	S

BSI, bloodstream infection; HAP/VAP, hospital-acquired/ventilator-associated pneumonia; UTI, urinary tract infection; AMK, amikacin; ATM, aztreonam; FEP, cefepime; CZA, ceftazidime-avibactam (avibactam at fixed concentration of 4 mg/L); C/T, ceftolozane-tazobactam (tazobactam at fixed concentration of 4 mg/L); CST, colistin; IPM, imipenem; I-R, imipenem-relebactam (relebactam at fixed concentration of 4 mg/L); MEM, meropenem; MVB, meropenem-vaborbactam (vaborbactam at fixed concentration of 8 mg/L); TZP, piperacillin-tazobactam (tazobactam at fixed concentration of 4 mg/L); TOB, tobramycin; FDC, cefiderocol.

Table S2. SNPs matrix of the ten *P. aeruginosa* ST111 isolates.

	PSA3_C2_US	PSA4_C2_US	PSA14_C2_BS	PSA49_C4_BS	PSA51_C4_BS	PSA62_C4_BS	PSA70_C4_RS	PSA71_C4_RS	PSA72_C4_RS	PSA73_C4_US
**PSA3_C2_US**	0	122	113	121	117	132	118	126	138	121
**PSA4_C2_US**	122	0	36	57	41	56	42	53	60	77
**PSA14_C2_BS**	113	36	0	69	33	50	36	43	56	69
**PSA49_C4_BS**	121	57	69	0	52	67	43	56	73	44
**PSA51_C4_BS**	117	41	33	52	0	17	21	30	23	54
**PSA62_C4_BS**	132	56	50	67	17	0	36	39	6	43
**PSA70_C4_RS**	118	42	36	43	21	36	0	23	40	53
**PSA71_C4_RS**	126	53	43	56	30	39	23	0	45	62
**PSA72_C4_RS**	138	60	56	73	23	6	40	45	0	49
**PSA73_C4_US**	121	77	69	44	54	43	53	62	49	0

BS, blood sample; RS, respiratory sample; US, urinary sample.

Table S3. SNPs matrix of the six *P. aeruginosa* ST235 isolates.

	**PSA1_C2_RS**	**PSA7_C2_RS**	**PSA8_C2_RS**	**PSA9_C2_US**	**PSA39_C5_RS**	**PSA41_C5_BS**
**PSA1_C2_RS**	0	34	198	498	272	312
**PSA7_C2_RS**	34	0	188	482	280	320
**PSA8_C2_RS**	198	188	0	446	250	290
**PSA9_C2_US**	498	482	446	0	474	514
**PSA39_C5_RS**	272	280	250	474	0	60
**PSA41_C5_BS**	312	320	290	514	60	0

BS, blood sample; RS, respiratory sample; US, urinary sample.
